# Esophageal bottle cap impaction in adult: A case report

**DOI:** 10.1097/MD.0000000000046538

**Published:** 2025-12-26

**Authors:** Haibo Liu, Guangchuan Shen, Kun Luo

**Affiliations:** a Department of Neurosurgery, The First Affiliated Hospital of Xinjiang Medical University, Urumqi, China; b Department of Otolaryngology, The Second People’s Hospital of Xindu District, Chengdu, China.

**Keywords:** case report, dysphagia, esophageal foreign body, ethanol–nicotine co-use, rigid esophagoscopy

## Abstract

**Rationale:**

Adult esophageal foreign-body impaction is typically food-related, whereas impaction of large non-food objects is rare and usually associated with structural abnormalities or altered mental status. We present an unusual case of an alert adult with chronic ethanol–nicotine co-use who remained asymptomatic for 3 hours after ingesting a beer bottle cap, highlighting the potential for substance-related impairment of protective pharyngeal reflexes.

**Patient concerns:**

A 49-year-old man with a 20-year history of daily alcohol and tobacco use developed acute pharyngeal pain 3 hours after attending a banquet. He denied any psychiatric history or prior dysphagia.

**Diagnoses:**

Digital radiography and computed tomography revealed a 2.5-cm radiopaque foreign body at the thoracic inlet, 18 cm from the incisors, consistent with a bottle cap. No underlying structural lesion was identified.

**Interventions:**

Flexible endoscopic retrieval failed due to persistent upper-esophageal sphincter tone. Rigid esophagoscopy under general anesthesia was successfully performed, enabling en bloc extraction of the bottle cap with minimal mucosal abrasion.

**Outcomes:**

Post-extraction endoscopy showed only superficial mucosal erosions without perforation or stricture. The patient was discharged uneventfully and remained asymptomatic at 3-month follow-up.

**Lessons:**

Chronic ethanol–nicotine co-use may blunt pharyngeal sensitivity and delay symptom onset, increasing the risk of large non-food esophageal foreign-body impaction. Clinicians should maintain a high index of suspicion for foreign-body impaction in substance-using adults, even in the absence of immediate symptoms or structural disease.

## 1. Introduction

Esophageal foreign bodies demonstrate distinct epidemiological patterns across populations. While western cohorts show predominance of meat boluses with frequent (45%) underlying pathology (eosinophilic esophagitis, strictures), Chinese populations exhibit higher rates of osseous impactions (60.4% fish/poultry bones) often at cervical constriction sites^.[1–3]^ The cricopharyngeal sphincter – the narrowest esophageal segment – accounts for 50% to 75% of impactions.^[4]^

Adult esophageal foreign-body impaction (EFBI) typically manifests with immediate odynophagia, making delayed presentation with large objects exceptionally rare outside neuromuscular disorders. We present the first documented case of beer cap impaction in a neurologically intact adult, highlighting how chronic alcohol-tobacco use disrupts protective swallowing mechanisms through:1. Cortical suppression of voluntary swallowing initiation.2. Brainstem reflex arc impairment.3. Oropharyngeal sensory desensitization.

## 2. Case presentation

**Figure 1.**
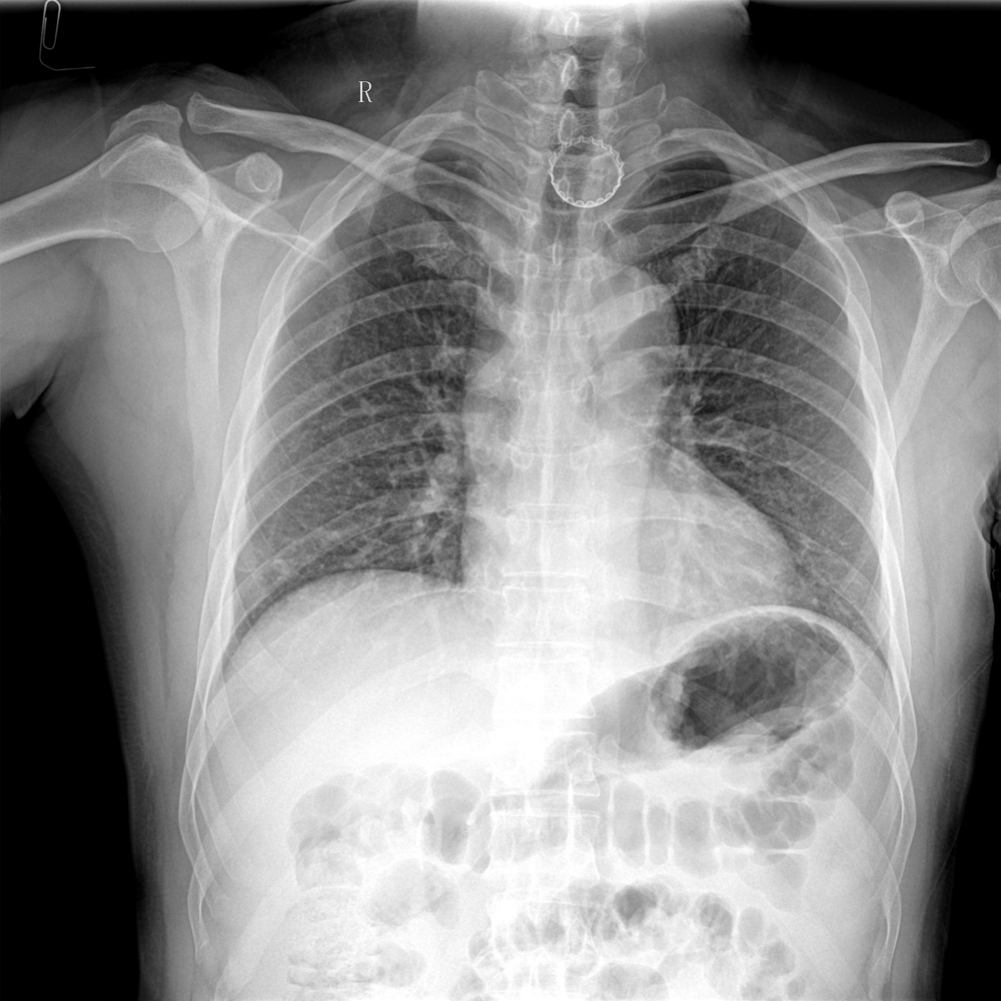
Digital radiography image revealed circular radiopacity at T1-T2 level (arrow).

**Figure 2.**
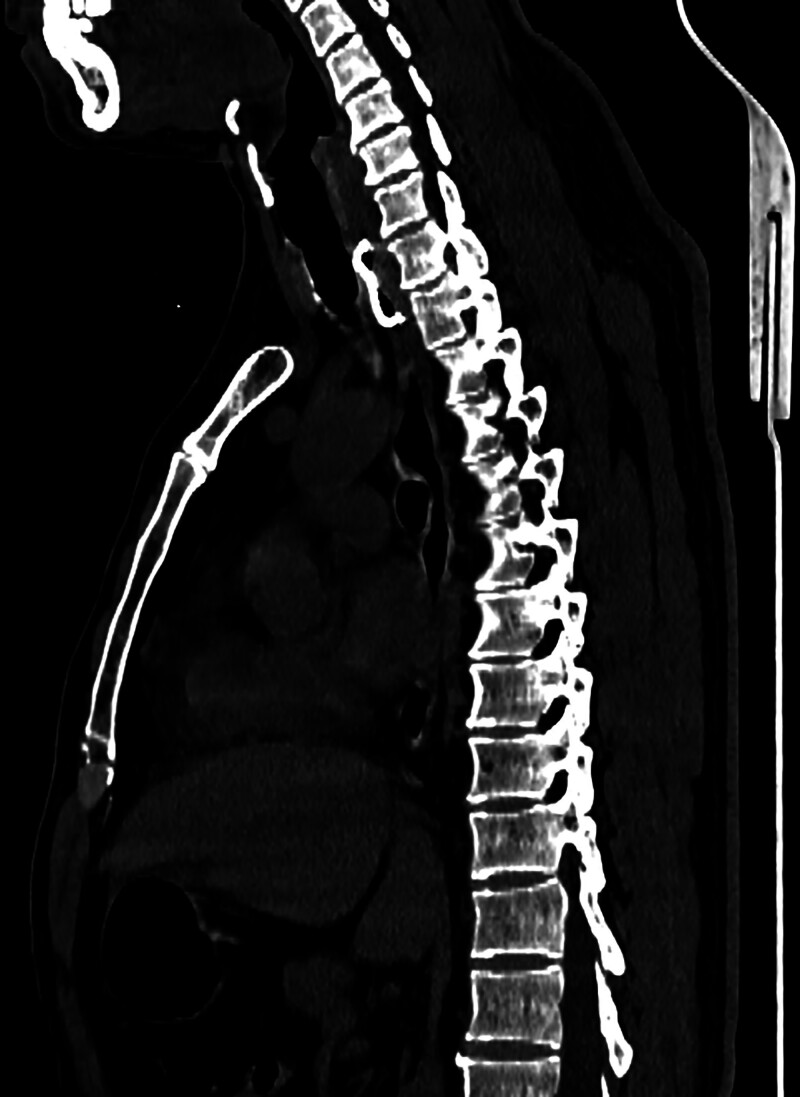
Axial, sagittal, and coronal computed tomography images revealed the foreign body to be round and flat, located slightly to the left of the anterior margin of the T1–T2 vertebral (arrow).

**Figure 3.**
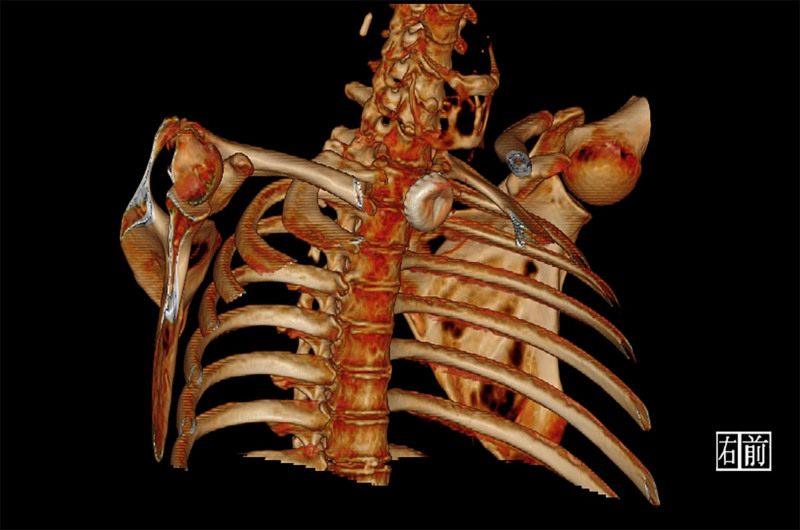
Chest CT 3-dimensional reconstruction demonstrated a metallic bottle cap impacted anterior to the T1–T2 vertebral bodies, with central concavity of the bottle cap (arrow). CT = computed tomography.

A 49-year-old male developed acute pharyngeal pain 3 hours after leaving a banquet (around 03:00). Because symptom onset occurred overnight, he presented to our emergency department the following morning. On arrival the patient was alert and oriented; no serum ethanol level was obtained. The patient reported a 20-year history of Ethanol consumption and nicotine use but denied recent foreign-body ingestion or personal/family history of psychiatric disorders. Indirect laryngoscopy revealed hyperemia and mild edema on the lingual surface of the epiglottis. Subsequent digital radiography and computed tomography imaging demonstrated a bottle cap-shaped radiopaque density at the thoracic inlet of the esophagus, suggestive of a foreign body (Figs. 1–3).

**Figure 4.**
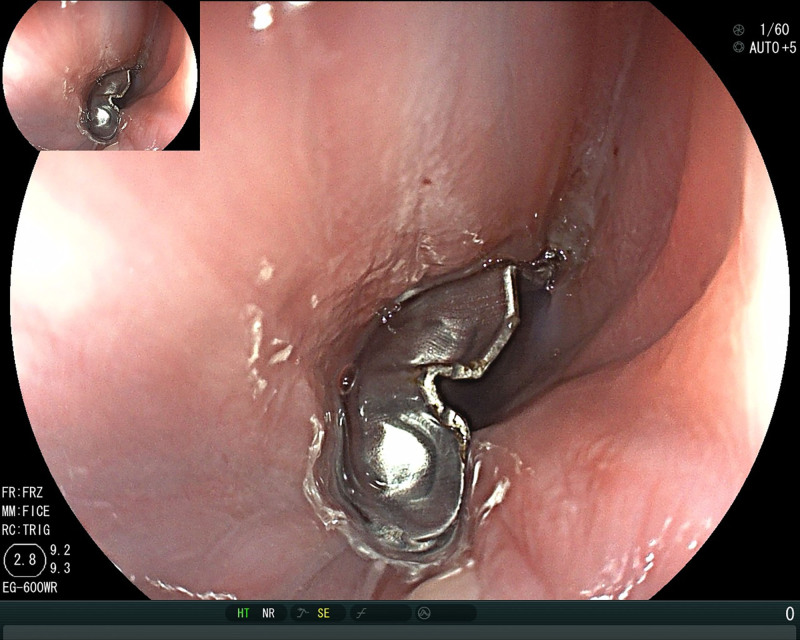
Gastroscopy revealed a retained metallic foreign body 20 cm from the incisors, with minor mucosal lacerations on the esophageal wall.

**Figure 5.**
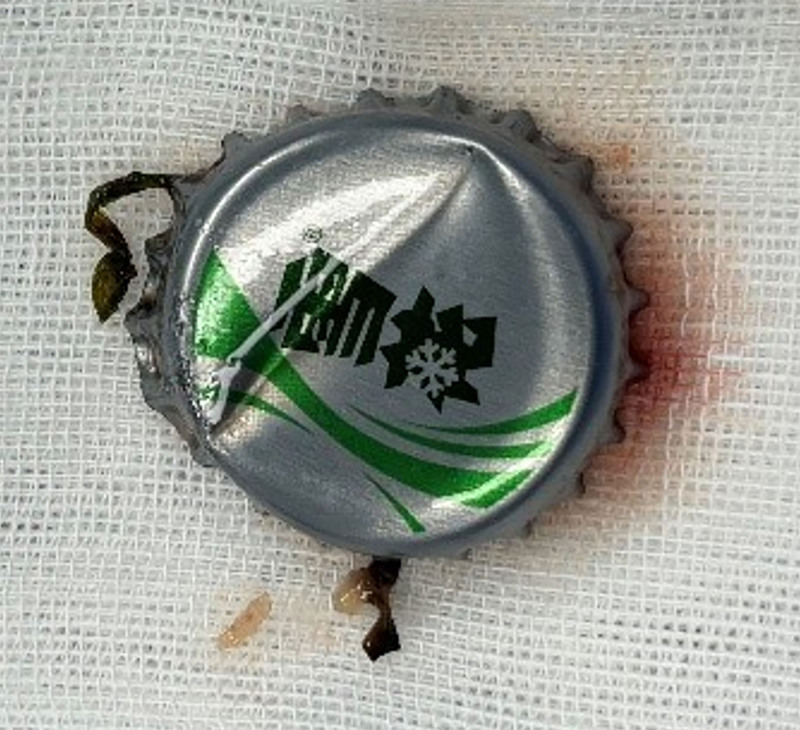
A rigid esophagoscope was used to successfully retrieve a 2.5 × 2.5 cm beer bottle cap, which featured the brand logo *“Snow.”*

Following multidisciplinary team evaluation, the patient was transferred to the endoscopic center for foreign-body extraction under flexible endoscope (Fig. 4). Owing to persistent upper-esophageal sphincter (UES) tone and the irregular geometry of the metallic foreign body, repeated transoral attempts with a flexible endoscope failed to advance the object through the UES. To avert additional esophageal trauma, written informed consent was obtained and rigid esophagoscopy was performed under propofol-based general anesthesia. The rigid esophagoscopy was introduced without resistance; the foreign body was grasped with an alligator forceps, apposed to the distal tip, and withdrawn en bloc with the endoscope. The radial stiffness of the rigid shaft provided continuous UES dilation, allowing atraumatic extraction of a 2.5 cm × 2.5 cm beer bottle cap bearing the *Snow* logo (Fig. 5). Post-extraction examination revealed minor esophageal wall abrasions with minimal bleeding. A nasogastric tube was placed for nutritional support. Notably, the retrieved “Snow” brand cap matched the beer consumed during the patient’s banquet earlier that evening, though he remained asymptomatic for hours post-ingestion.

**Figure 6.**
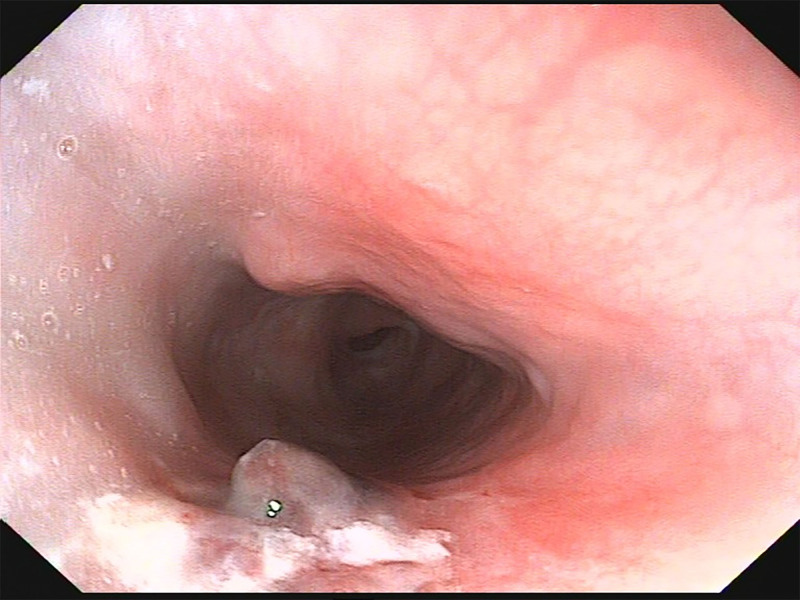
On the third postoperative day, gastroscopy revealed a linear erosion approximately 5 mm in length between 20 cm from the incisors and the esophageal inlet, as well as a mucosal elevation about 5 mm in size with a surface consistent with the surrounding mucosa. A similar-sized blood-crusted elevation was observed on the contralateral side, with surrounding mucosa showing significant erosion and easy bleeding on contact. The remaining mucosa appeared smooth and moist with clear vascular markings. The cardia was well-closed with smooth mucosa.

On postoperative day 3, the patient self-removed the nasogastric tube due to pharyngeal discomfort. Follow-up endoscopy showed: (1) Linear erosions (5mm) and mucosal elevations (5mm diameter) 20cm from incisors to the esophageal inlet. (2) Contralateral hematoma-like protrusions with significant erosive changes and contact bleeding (Fig. 6).

During 3-month surveillance, no pharyngeal symptoms or proximal esophageal strictures were observed. Continued long-term monitoring remains ongoing. The reporting of this study conforms to the Case Report (CARE) guidelines.^[5]^

## 3. Discussion

Overall, the majority of foreign-body ingestions occur in the pediatric population, with coins and toys being the most commonly swallowed objects.^[2,3,6]^ In western cohorts, approximately 45% of patients with upper gastrointestinal foreign-body impactions have underlying medical conditions. These include esophageal strictures (12%), gastroesophageal reflux disease (10%), eosinophilic esophagitis (9%), Schatzki rings (7%), achalasia (5%), and malignant neoplasms (2%).^[1]^ Pediatric studies demonstrate that eosinophilic esophagitis is the leading cause of food impactions, accounting for as high as 53% of cases.^[7]^ The most frequently encountered impacted objects are nonmeat food items (31%), meat (25%), and coins (21%).^[1]^ However, due to ethnic and dietary differences, only 9.8% of foreign-body impactions in China are attributed to such underlying conditions. The predominant pathological causes are postoperative anastomotic strictures following esophageal cancer resection (30.2%) and esophageal strictures (23.3%). The most common impacted object is fish bones (60.4%), with the majority located in the upper esophagus (82.2%).^[8]^ Among adults, true foreign-body ingestion (nonfood objects) is more prevalent in patients with psychiatric disorders, developmental delays, ethanol intoxication, and incarcerated individuals seeking secondary gain through medical facilities.^[2]^

Due to physiological and pathological narrowing, most EFBIs occur at the most constricted segment of the upper esophagus, particularly at the cricopharyngeal muscle.^[9,10]^ Retrosternal pain, dysphagia, and odynophagia represent the most common clinical manifestations in adults.^[9]^ Major complications of EFBI include mucosal hemorrhage, erosion, ulceration, fistula formation, perforation, and gastrointestinal obstruction. Notably, impacted metallic objects are the leading cause of esophageal perforation.^[10]^

The primary treatment modalities for ingested upper gastrointestinal foreign bodies include spontaneous passage, endoscopic retrieval, and surgical intervention. Studies indicate that in western cohorts, approximately 80% or more of foreign bodies pass spontaneously without requiring medical intervention. However, in cases of intentional ingestion, the rate of endoscopic intervention is significantly higher (63%–76%), with up to 12% to 16% of patients ultimately requiring surgical management.^[2]^ In China, the epidemiological profile of upper gastrointestinal foreign bodies differs from that in western cohorts, resulting in a higher proportion of patients undergoing endoscopic procedures. For foreign bodies lodged in the proximal esophagus, either flexible gastroscopy or rigid esophagoscopy is considered the first-line approach.^[3]^ In the present case, initial attempts at retrieval via flexible endoscopy were unfortunately unsuccessful. The foreign body was ultimately removed under general anesthesia using rigid esophagoscopy. Compared to flexible gastroscopy, rigid esophagoscopy offers an expanded operative field and enhanced maneuverability for foreign-body extraction.

In normal adults, the retention of hard, irregularly shaped, and large foreign bodies like beer bottle caps in the esophagus is relatively uncommon, differing from typical EFBIs caused by food or coins. Li et al^[9]^ reported a case of prolonged esophageal foreign-body retention in an adult, primarily analyzing the treatment and clinical manifestations, but did not elaborate on the neural reflex mechanisms underlying the swallowing of such a conspicuously large object.

Swallowing is a complex reflex that depends on a highly programmed neuromuscular sequence. Functionally it is divided into 3 successive but distinct phases – oral, pharyngeal and esophageal – of which only the oral phase is under voluntary control; the pharyngeal and esophageal phases are governed automatically by the medullary swallowing center.^[11]^ At the suprabulbar level, the cerebral cortex and cerebellum modulate voluntary swallowing by setting reflex thresholds and by initiating and sequencing the entire motor program. Cortical descending pathways interact reciprocally with the brainstem swallowing network, permitting precise temporal regulation of reflex onset and coordination and thereby ensuring that swallowing proceeds efficiently and safely.^[11,12]^

Both ethanol and nicotine significantly impair central nervous system responses and pharyngeal protective reflexes, thereby increasing the risk of foreign-body aspiration. Ethanol potentiates the inhibitory effects of Gamma-Aminobutyric Acid type A (GABAₐ)receptors, suppressing neuronal activity in the medullary swallowing centers as well as the higher-order autonomic regulation mediated by the cerebral cortex and cerebellum.^[13–17]^ This inhibition compromises both the initiation and coordination of the swallowing reflex. Furthermore, ethanol diminishes the sensitivity of pharyngeal mechanoreceptors and impairs the ability of oral and pharyngeal mucosa to detect foreign bodies, further degrading protective pharyngeal reflexes.^[17]^ Studies demonstrate that a serum ethanol concentration of 5 to 10 mmol/L significantly enhances GABAA receptor activity, reduces excitatory neurotransmission, and induces cognitive dysfunction – including impaired attention, memory alterations, and somnolence – all of which elevate aspiration risk.^[17]^

Smoking also adversely affects protective swallowing reflexes, primarily through local irritation of the pharynx, which impairs pharyngeal sensory receptors – rather than via systemic nicotine effects.^[15]^ This localized impact is associated with smoking-induced regional inflammation, mucosal damage, and impaired neural signaling, thereby increasing the risk of aspiration.

There is a robust epidemiological association between ethanol and nicotine use.^[18,19]^ Evidence directly linking their concomitant consumption to an increased risk of foreign-body ingestion is currently lacking. however, experimental and clinical studies indicate that either agent alone can impair pharyngeal sensory discrimination and disrupt swallowing coordination, thereby predisposing to aspiration.^[15,20,21]^

The combined use of ethanol and nicotine – particularly combustible tobacco – can produce additive or even synergistic pathophysiological effects.^[22]^ This may offer a biologically plausible mechanism for the increased risk of foreign-body ingestion.

## 4. Conclusions

This case suggests that chronic ethanol–nicotine co-use may contribute to delayed presentation of large, nonfood foreign bodies via GABAA receptor potentiation and oropharyngeal mechanoreceptor desensitization; clinicians should therefore maintain high suspicion for EFBI in substance-using patients even when symptom onset is delayed.

## Acknowledgments

The authors would like to acknowledge Jianbo Lei, MD for his help in the computed tomography 3-dimensional reconstruction of this report. This report is supported by the Leading Talent Project (Development and Application of Detection Instrument for Analysis of Glioma Cells at Different Grades Based on Deconvolution Combined with Gene Expression, No. 2023TSYCLJ0030).

## Author contributions

**Conceptualization:** Haibo Liu, Kun Luo.

**Data curation:** Guangchuan Shen.

**Resources:** Guangchuan Shen.

**Writing – original draft:** Haibo Liu.

**Writing – review & editing:** Kun Luo.

## References

[1] Sperry SL Crockett SD Miller CB Shaheen NJ Dellon ES. Esophageal foreign-body impactions: epidemiology, time trends, and the impact of the increasing prevalence of eosinophilic esophagitis. Gastrointest Endosc. 2011;74:985–91. 21889135 10.1016/j.gie.2011.06.029 PMC3951006

[2] Ikenberry SO Jue TL Anderson MA; ASGE Standards of Practice Committee. Management of ingested foreign bodies and food impactions. Gastrointest Endosc. 2011;73:1085–91. 21628009 10.1016/j.gie.2010.11.010

[3] Lin JH Fang J Wang D; Chinese Society of Digestive Endoscopy. Chinese expert consensus on the endoscopic management of foreign bodies in the upper gastrointestinal tract (2015, Shanghai, China). J Dig Dis. 2016;17:65–78. 26805028 10.1111/1751-2980.12318

[4] Dray X Cattan P. Foreign bodies and caustic lesions. Best Pract Res Clin Gastroenterol. 2013;27:679–89. 24160927 10.1016/j.bpg.2013.08.009

[5] Gagnier JJ Kienle G Altman DG Moher D Sox H Riley D; CARE Group. The CARE guidelines: consensus‐based clinical case reporting guideline development. Headache. 2013;53:1541–7. 24228906 10.1186/1752-1947-7-223 PMC3844611

[6] Birk M Bauerfeind P Deprez P. A. removal of foreign bodies in the upper gastrointestinal tract in adults: European Society of Gastrointestinal Endoscopy (ESGE) clinical guideline. Endoscopy. 2016;48:489–96. 26862844 10.1055/s-0042-100456

[7] Diniz LO Towbin AJ. Causes of esophageal food bolus impaction in the pediatric population. Dig Dis Sci. 2012;57:690–3. 21948341 10.1007/s10620-011-1911-8

[8] Zhang S Cui Y Gong X Gu F Chen M Zhong B. Endoscopic management of foreign bodies in the upper gastrointestinal tract in South China: a retrospective study of 561 cases. Dig Dis Sci. 2010;55:1305–12. 19655249 10.1007/s10620-009-0900-7

[9] Li Y Wang R Feng Q Zhang S Wang C Song X. Long-term retainment of a foreign body in the esophagus in an adult: a case report. J Int Med Res. 2023;51:3000605231152392. 36794554 10.1177/03000605231152392 PMC9936534

[10] Wang X Zhao J Jiao Y Wang X Jiang D. Upper gastrointestinal foreign bodies in adults: a systematic review. Am J Emerg Med. 2021;50:136–41. 34365062 10.1016/j.ajem.2021.07.048

[11] Hamdy S Aziz Q Rothwell JC. Explaining oropharyngeal dysphagia after unilateral hemispheric stroke. Lancet (London, England). 1997;350:686–92. 9291902 10.1016/S0140-6736(97)02068-0

[12] Hamdy S Aziz Q Rothwell JC. The cortical topography of human swallowing musculature in health and disease. Nat Med. 1996;2:1217–24. 8898748 10.1038/nm1196-1217

[13] Carta M Mameli M Valenzuela CF. Alcohol enhances GABAergic transmission to cerebellar granule cells via an increase in golgi cell excitability. J Neurosci. 2004;24:3746–51. 15084654 10.1523/JNEUROSCI.0067-04.2004 PMC6729340

[14] Aguayo L Peoples R Yeh H Yevenes G. GABA-a receptors as molecular sites of ethanol action. Direct or indirect actions? Curr Top Med Chem. 2002;2:869–85. 12171577 10.2174/1568026023393426

[15] Dua KS Surapaneni SN Santharam R Knuff D Hofmann C Shaker R. Effect of Systemic Alcohol and Nicotine on Airway Protective Reflexes. Am J Gastroenterol. 2009;104:2431–8. 19550414 10.1038/ajg.2009.330 PMC4160881

[16] Wallner M Hanchar HJ Olsen RW. Low dose acute alcohol effects on GABAA receptor subtypes. Pharmacol Ther. 2006;112:513–28. 16814864 10.1016/j.pharmthera.2006.05.004 PMC2847605

[17] Liang J Olsen RW. Alcohol use disorders and current pharmacological therapies: the role of GABAA receptors. Acta Pharmacol Sin. 2014 ;35:981–93. 25066321 10.1038/aps.2014.50 PMC4125717

[18] Garnett C Oldham M Brose L Cheeseman H Cox S. Prevalence and characteristics of co-occurrence of smoking and increasing-and-higher-risk drinking: a population survey in England. Addict Behav. 2024;150:107928. 38091779 10.1016/j.addbeh.2023.107928

[19] Beard E West R Michie S Brown J. Association between smoking and alcohol‐related behaviours: a time–series analysis of population trends in England. Addiction. 2017;112:1832–41. 28556467 10.1111/add.13887 PMC5600127

[20] Dua K Bardan E Ren J Sui Z Shaker R. Effect of chronic and acute cigarette smoking on the pharyngo-upper oesophageal sphincter contractile Reflex and Reflexive pharyngeal swallow. Gut. 1998;43:537–41. 9824582 10.1136/gut.43.4.537 PMC1727281

[21] Dua K Bardan E Ren J Sui Z Shaker R. Effect of chronic and acute cigarette smoking on the pharyngoglottal closure reflex. Gut. 2002;51:771–5. 12427774 10.1136/gut.51.6.771 PMC1773456

[22] Jackson SE Oldham M Garnett C Brown J Shahab L Cox S. Smoking, and to a lesser extent non-combustible nicotine use, is associated with higher levels of alcohol consumption and risky drinking. Sci Rep. 2025;15:6851. 40011541 10.1038/s41598-025-89750-2 PMC11865552

